# Foodborne Disease Risk Prediction Using Multigraph Structural Long Short-term Memory Networks: Algorithm Design and Validation Study

**DOI:** 10.2196/29433

**Published:** 2021-08-02

**Authors:** Yi Du, Hanxue Wang, Wenjuan Cui, Hengshu Zhu, Yunchang Guo, Fayaz Ali Dharejo, Yuanchun Zhou

**Affiliations:** 1 Computer Network Information Center Chinese Academy of Sciences Beijing China; 2 Chinese Academy of Sciences University Beijing China; 3 Baidu Inc Beijing China; 4 China National Center for Food Safety Risk Assessment Beijing China

**Keywords:** foodborne disease, risk, prediction, spatial–temporal data

## Abstract

**Background:**

Foodborne disease is a common threat to human health worldwide, leading to millions of deaths every year. Thus, the accurate prediction foodborne disease risk is very urgent and of great importance for public health management.

**Objective:**

We aimed to design a spatial–temporal risk prediction model suitable for predicting foodborne disease risks in various regions, to provide guidance for the prevention and control of foodborne diseases.

**Methods:**

We designed a novel end-to-end framework to predict foodborne disease risk by using a multigraph structural long short-term memory neural network, which can utilize an encoder–decoder to achieve multistep prediction. In particular, to capture multiple spatial correlations, we divided regions by administrative area and constructed adjacent graphs with metrics that included region proximity, historical data similarity, regional function similarity, and exposure food similarity. We also integrated an attention mechanism in both spatial and temporal dimensions, as well as external factors, to refine prediction accuracy. We validated our model with a long-term real-world foodborne disease data set, comprising data from 2015 to 2019 from multiple provinces in China.

**Results:**

Our model can achieve F1 scores of 0.822, 0.679, 0.709, and 0.720 for single-month forecasts for the provinces of Beijing, Zhejiang, Shanxi and Hebei, respectively, and the highest F1 score was 20% higher than the best results of the other models. The experimental results clearly demonstrated that our approach can outperform other state-of-the-art models, with a margin.

**Conclusions:**

The spatial–temporal risk prediction model can take into account the spatial–temporal characteristics of foodborne disease data and accurately determine future disease spatial–temporal risks, thereby providing support for the prevention and risk assessment of foodborne disease.

## Introduction

Foodborne disease is caused by pathogenic bacteria that enter the body due to ingestion of contaminated food, resulting in symptoms such as diarrhea and abdominal pain [1]. According to the World Health Organization, more than 600 million people worldwide suffer from diseases caused by contaminated food every year, of whom 4.2 million die of foodborne illness [2]. The high incidence of foodborne diseases seriously threatens health and social economy. Most existing research efforts on foodborne disease have mostly been concentrated in the fields of medical science and food safety [3-6]; however, researchers have turned their attention to exploiting machine learning technologies to address foodborne disease–related topics, such as analyzing the correlation between foodborne diseases and food [7], discovering foodborne disease outbreak locations using social media [8-10], analyzing foodborne disease pathogens [11,12], and predicting foodborne disease outbreaks [13-15]. While considerable efforts have been made, an open challenge remains—accurately predicting foodborne disease risk by mining spatial–temporal patterns in historical disease records, using similar methods to those used for flu prediction [16-18], which is of great significance for public health management. By providing estimates of the trends of foodborne disease in future periods, accurate foodborne disease risk prediction can support effective guidance for government epidemic prevention policies. Because foodborne disease risk usually follows a certain spatial–temporal pattern—for example, the incidence in summer is higher than those in autumn and winter, and risk of foodborne diseases in a region is similar to those in regions with similar weather or urban functional structure—the prediction of foodborne disease risk can be solved as a spatial–temporal data modeling problem.

In the literature, a variety of methods for spatial–temporal data modeling have been proposed, including traditional statistical models [19,20] and deep learning methods, such as recurrent neural network [21], long short-term memory (LSTM) [22], convolutional neural network [23], graph convolutional network [24], temporal graph convolutional network [25], and structural recurrent neural network [26]. To solve the problem of spatial–temporal data modeling, structural recurrent neural networks use recurrent neural networks to model temporal dependence and model spatial dependence with structural recurrent neural networks on spatial–temporal graphs. Such models possess scalability; however, models are limited to static representations of spatial dependence by region proximity (ie, the models lack dynamic spatial correlation representation).

Compared with COVID-19 [27], influenza [16-18], and other infectious diseases [28], foodborne disease is spread through food rather than people. Therefore, the data characteristics of foodborne disease outbreaks are quite different from those related to infectious diseases, for example, sparse data increase the difficulty of predicting foodborne disease risk. Foodborne disease risk prediction also differs from traffic prediction [25,29-33]. Traffic problems require short-term prediction, while foodborne disease risk problems require long-term prediction.

To address these challenges, in this paper, we propose the use of a multigraph structural LSTM based spatial–temporal prediction model to determine the risk of foodborne disease in different regions in future periods, which considers various spatial dependencies and uses a dynamic fusion method, with multistep prediction using a encoder–decoder structure, to support future disease prevention and control, and with attention mechanisms in spatial and temporal dimensions, as well as external features, to further improve performance. To the best of our knowledge, this is the first study to focus on spatial–temporal foodborne disease risk prediction and report validation results using real-world data sets.

We propose a multistep spatial–temporal data prediction model based on encoder–decoder structure and composed entirely of LSTM modules, to address the problem of spatial–temporal foodborne disease risk prediction; we propose a dynamic fusion method to fuse region proximity, historical trend similarity, regional function similarity and food exposure similarity, with a spatial–temporal attention mechanism and external feature embedding; and we validated our model with extensive experiments on a long-term real-world foodborne disease data set, with data from 2015 to 2019 in multiple provinces of China; experimental results clearly demonstrated that our approach can outperform other state-of-the-art methods, with a margin.

## Methods

### Problem Definition

#### Region Graph

We divide each city or region into irregular subregions by administrative areas and organized them into an undirected graph ***G***=(***v***, ***e***, *A*), where *v* is a set of nodes and each node corresponds to a subregion, *e* is a set of edges with each edge connecting 2 subregions defined by some rules, and *A* represents the adjacency matrix of ***G***. In particular, each *v_i_* in *v*=(*v_1_*, *v_2_*,...*v_n_*) is the minimal spatial unit, where *N* is the total number of spatial units, and *e_ij_* is the edge that connects *v_i_* and *v_j_*.

#### Historical Data Sequence

To represent the historical data sequence, we calculated the number of disease records at each prediction period, that is, given a subregion *v_i_*, we defined the sequence of counts 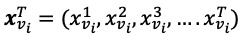 to denote the historical data sequence in subregion *v_i_* during the time window *T*.

#### Spatial–Temporal Graph

To represent spatial–temporal data characteristics, we organized the historical data sequence and the spatial graph into spatial–temporal graphs. Foodborne disease data at timestep *t* in a subregion is represented as graph signal 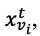, and the entire spatial–temporal graph is represented as 
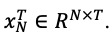


#### Disease Risk

To evaluate the predicted disease risk intuitively, we divided each region’s disease record count into 2 classes using a ratio, which we determined by consulting domain experts: when the disease record count in a region at any given timestep exceeds 70% of the historical sequence of this region, the risk at that timestep for that region is considered high risk or low risk.

#### Disease Risk Prediction

The risk of foodborne disease in a region is affected by its historical data and by the risk of surrounding area and is, therefore, a spatial–temporal prediction problem. Given the historical disease record data from subregions *v* during time period ***T***, our task was to determine the unknown disease risk level for each subregion in future time slots ***L***. Formally, our aim was to compute the following:


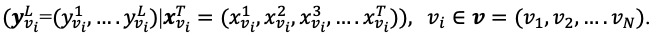


### Model Framework

#### Model Overview

**Figure 1:**
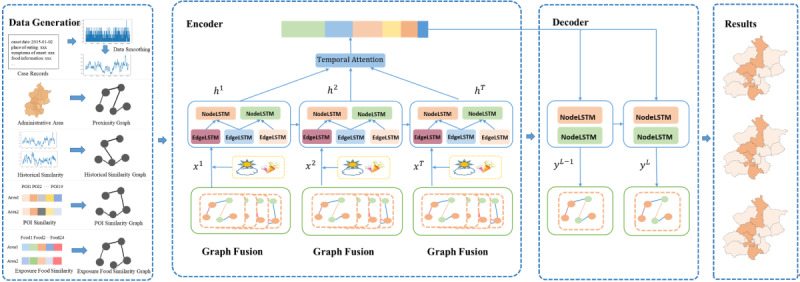
Foodborne disease spatial–temporal risk prediction model framework. LSTM: long short-term memory; POI: point of interest.

Our model is an encoder–decoder multigraph structural LSTM (Figure 1). This model consists of 5 modules. The *Data Generation* module comprises temporal sequence and multiple spatial graph (geographic proximity, historical data similarity, regional functional similarity, and foodborne disease exposure food similarity) data processing. The *Multigraph Fusion* module takes into account multiple spatial correlations and merges them dynamically. The *Encoder–Decoder* module uses LSTM networks to model temporal dependence and spatial dependence of foodborne disease risk by using the edge LSTM and the node LSTM, respectively, simultaneously in the encoder. In the decoder, the node LSTM is used to predict foodborne disease risk in each region in the 1 or more future timesteps. The *Spatial–Temporal Attention* module takes spatial–temporal relationship complexity into account and assigns temporal importance values to timesteps and spatial importance values to adjacent edges of nodes. The *External Feature Embedding* module combines various external features (eg, holidays, temperature) and merges external features into the encoder at each timestep.

#### Data Generation

This module performs data processing of temporal sequence and multiple spatial graph data (geographic proximity, historical data similarity, regional functional similarity, and foodborne disease exposure food similarity).

Temporal sequence data were collected from historical foodborne disease records, from which disease record counts were calculated. Due to the sparseness of data, we performed data augmentation, with a sliding 1-month window by moving the start of the unnatural month, which resulted in an expansion of the data. Temporal sequence data were normalized (range 0-1), using minimum–maximum normalization.

Data were characterized by regional proximity because, intuitively, adjacent regions will have similarity risks of disease due to climate and geography, as well as from population movement between regions. For graph ***G***=(***v***, ***e***, *A*), if *v_i_* and *v_j_* are spatially adjacent, then 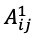 is 1, otherwise is 0.


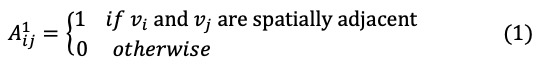


For each region, disease risk trends will follow a relatively fixed pattern, and regions with similar historical disease risk trends will have similar disease risk trends in future periods. We used historical data sequence to calculate the pairwise historical similarities between regions using Pearson correlation coefficients. We set a threshold; the adjacency value 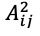 between 2 nodes *v_i_* and *v_j_* with a similarity less than the threshold is 0. The threshold is used to control the sparsity of edges.


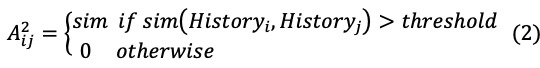


Regions with similar urban functions will have similar population and business structures, and thus, similar foodborne disease risk. We used point-of-interest (POI) data from each region to characterize this feature. POI can be divided by function into 19 categories, the term frequency–inverse document frequency can be used to embed these data as vectors for every region, and the similarity between of POI vectors for regions can be evaluated [34].


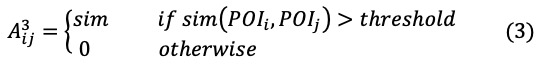


Exposure food, the transmission medium of foodborne disease, plays an important role in the prediction of foodborne disease risk. Intuitively, exposure to foodborne diseases at different timesteps and in different regions are different, and the impact on the risk of foodborne diseases is also different. Therefore, we counted the number of exposures for each food category (23 categories) in different regions at different timesteps, which were represented as vectors using term frequency–inverse document frequency. Similarities between exposure vectors for regions at each timestep were calculated, representing spatial correlations.


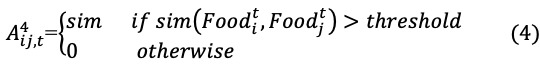


#### Multigraph Fusion

Our dynamic fusion method, for multiple spatial graphs constructed by different spatial correlations, was designed to merge adjacent matrices {*A^1^*, *A^2^*...*A^m^*}, where *m* represents the number of constructed graphs. We defined 4 parameters, *W_1_*, *W_2_*, *W_3_*, *W_4_*, and to obtain the dynamic merged graph, element-wise products between the parameters and adjacent matrices tare calculated to adjust the weights of the geographic proximity, historical data similarity, region functional similarity, and exposure food similarity graphs.


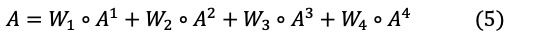


The parameters are continuously adjusted, through network learning, to control the influences of multiple spatial dependencies on the final inputs.

#### Encoder–Decoder

In order to model spatial dependence and temporal dependence simultaneously and conduct multistep prediction, we organize the historical temporal sequence data and the fused spatial graph into the structure of spatial–temporal graph and construct a graph structural LSTM model of encoder–decoder architecture inspired by the structural recurrent neural network architecture [26].

**Figure 2:**
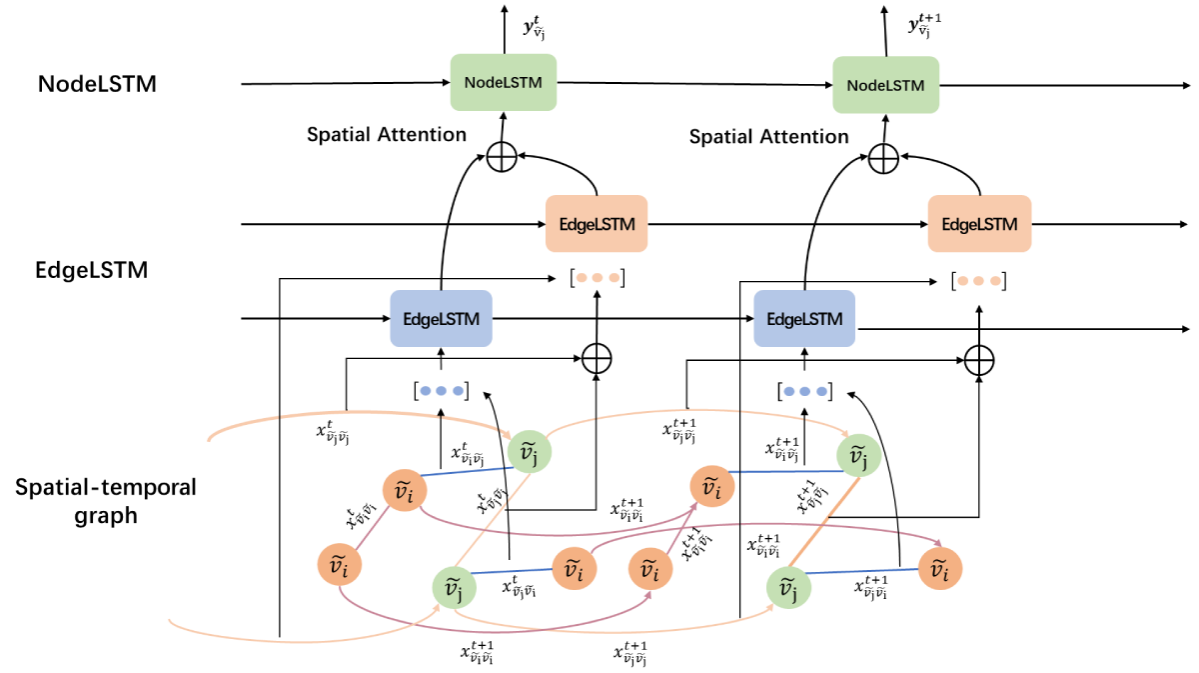
Structural long short-term memory (LSTM) details.

**Figure 3:**
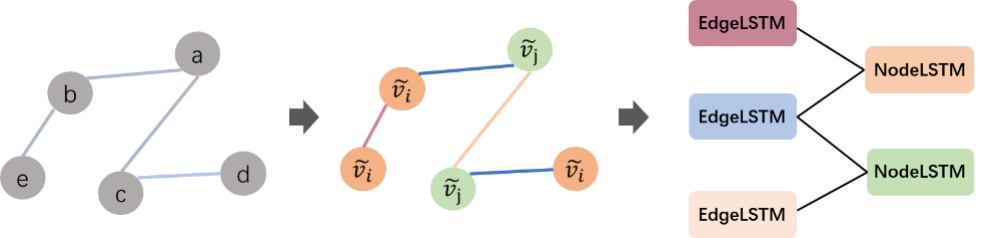
Grouping nodes and edges in long short-term memory (LSTM) networks.

In the encoder, a structural LSTM network (Figure 2) was constructed with node LSTMs and edge LSTMs to model temporal dependence and spatial dependence. We divide nodes *v*=(*v_1_*, *v_2_*,...*v_n_*) on the spatial graph into 2 categories 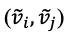 in a ratio according to the sum of values of each node at all timesteps in the temporal dimension. The edges between nodes were divided into 3 categories, according to connected nodes. Then, we constructed node LSTMs and edge LSTMs for each category of nodes and each category of edges (Figure 3). For each edge LSTM, the input at each timestep was the concatenation of the current node values connected by the edges of its category, and for each node LSTM, the input at each timestep was the fusion of the current outputs of edge LSTMs related to its node category. It not only contained the information of the current category of nodes but also contained the information of adjacent node categories to model spatial dependence. The current state of the node LSTM and edge LSTM was not only influenced by the current input, but also by the previous timesteps, to model temporal dependence.

In the decoder, for each node LSTM, we used the context vector learned from the encoder to predict the value of 1 or more timesteps in the future.

#### Spatial-Temporal Attention

In order to eliminate the influence of distance on temporal dependence, and to fully consider temporal and spatial correlations, we applied a spatial-temporal attention mechanism. In the temporal dimension, we calculate the score between hidden states with current spatial–temporal state, transformed into a normalized value with softmax operation, then apply a weighted summarization as


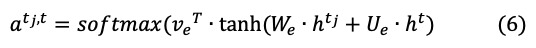



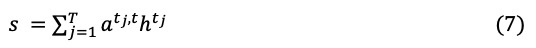


In the spatial dimension, we calculate the score of each edge LSTM, normalized by softmax to assign different weight to different edge LSTM every timestep.

#### External Feature Embedding

The risk of foodborne disease may be influenced by the change of external factors (for example, people eating out on holidays more often than working days, or high temperature and humid weather being more likely to cause food spoilage). Therefore, to incorporate external features into our model, we first preprocess temperature data by filling the missing value and computing the mean value for a month. For the holiday feature, we calculated the number of holidays per month, which was represented as a series of fixed-length vectors and concatenated 
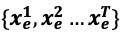
 with the input sequence of node LSTMs in previous timesteps to predict the future disease risk.

### Model Validation

#### Data Set

We validated our model using a real-world data set (China National Center for Food Safety Risk Assessment [35]), which consisted of foodborne disease records reported by sentinel hospitals in almost all provinces in China. Each record contains information such as time of onset, place of eating, place of living, symptoms of onset, and food information. We selected all the records in the 4 provinces with best-quality data from 2015 to 2019—Beijing, Zhejiang, Shanxi, and Hebei. Due to data acquisition limitations, we only obtain the POI information for Beijing. Therefore, only 3 spatial dependencies were used for Zhejiang, Shanxi, and Hebei. We collected temperature data and holiday data from 2015 to 2019 to simulate the impact of weather and holiday on the foodborne disease risk.

### Comparison Models and Evaluation Metrics

We compared our model with historical average, autoregressive, ARIMA (autoregressive integrated moving average), LSTM, and spatial–temporal graph convolutional network models. Historical average models estimate future results by computing the average value of historical data, which is too simple to model spatial-temporal dependence. Autoregressive models are statistical time-series models that use a linear combination of the values of several previous timesteps to describe future values. ARIMA models, which as the name implies, use autoregressive terms and moving average terms. Data must be processed before applying the ARIMA model to ensure that data are stationary. LSTM networks are mostly used for natural language processing problems [22]. LSTM networks can learn sequence dependence due to its chain structure. We applied LSTM to every node of the graph and evaluated the model by merging the results of all nodes. Spatial–temporal graph convolutional network models are based on convolutional neural networks but use graph convolutional networks instead of traditional convolutional neural networks for spatial dimensions and temporal convolutional neural network instead of recurrent neural networks for temporal dimensions. Spatial–temporal graph convolutional network models have achieved outstanding results in traffic prediction [31].

Given that we used a binary definition of disease risk, to avoid the effect of imbalances between 2 classes, we used


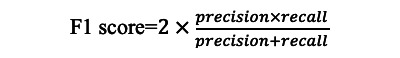


to evaluate model performance. In order to avoid the effect of parameter initialization on the results, we performed 5 trials for each model and averaged the results.

## Results

### Performance Comparison

#### Comparison With Other Methods

**Figure 4:**
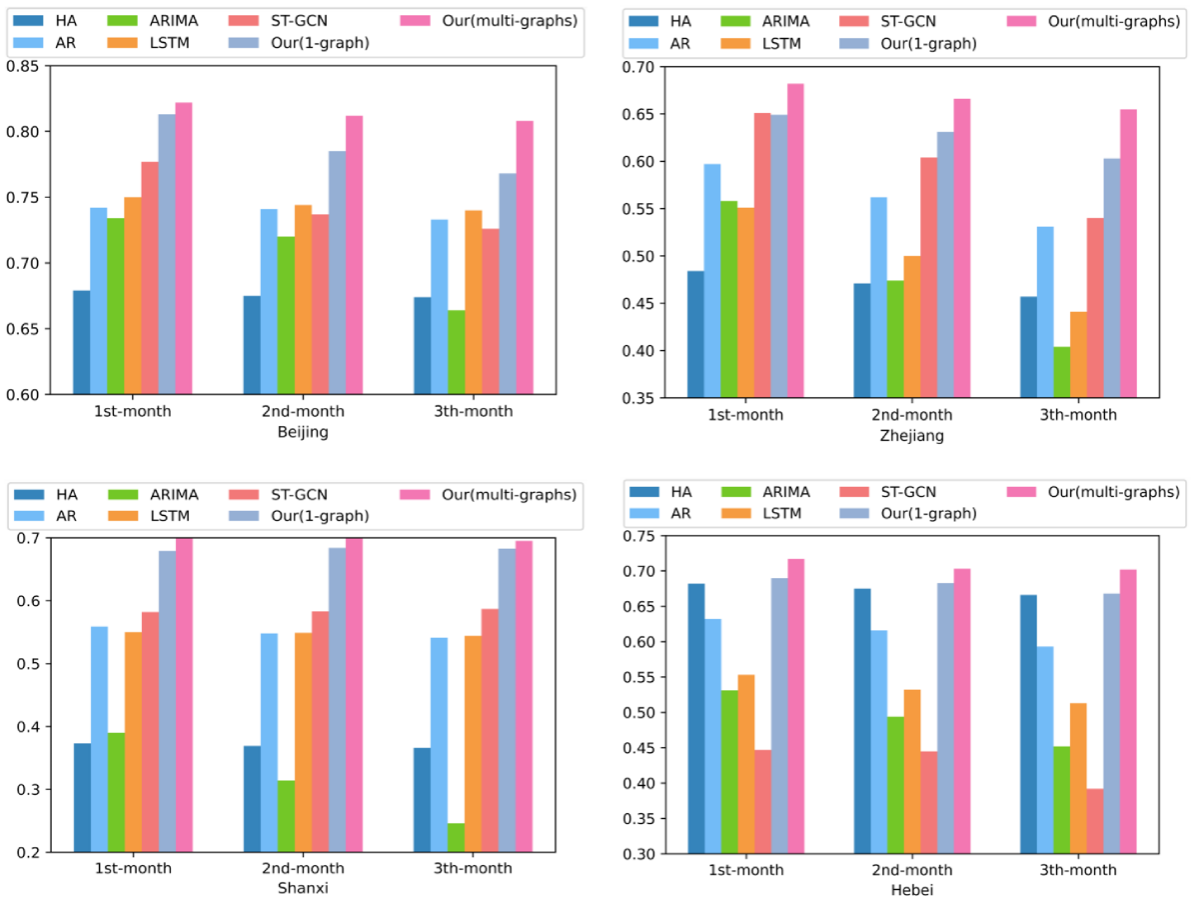
Performance in 4 provinces. AR: autoregressive; ARIMA: autoregressive integrated moving average; HA: historical average; LSTM: long short-term memory; ST-GCN: spatial–temporal graph convolutional network.

Table 1 and Figure 4 summarize foodborne disease risk prediction performance results for 1, 2, and 3 months in each of the 4 provinces. Our proposed model outperformed all other models for all 4 provinces and achieved the highest F1 score for every forecast period. Traditional statistical models, such as autoregressive and ARIMA models, performed worse than deep learning models for most provinces, indicating that traditional methods were too simple to solve complex nonlinear spatiotemporal problems. LSTM networks modeled the temporal dependence of each node on the spatial–temporal graph independently and ignored the dynamic spatial correlation between nodes, resulting in relatively poor performance. The spatial–temporal graph convolutional network model used convolution neural networks to model temporal dependence as well as spatial dependence, with better performance than that of the LSTM model for most provinces. Our proposed method with a single graph (that is, a regional proximity graph) simulated temporal dependence and spatial dependence simultaneously with a reasonable attention mechanism, resulting in better performance than those of the other methods. At most timesteps, it had the second-best prediction results. By accounting for rich spatial dependencies, our multigraph model exhibited better performance than that of the single-graph model for all 4 provinces, achieving the best results. The highest F1 score was 20% higher than the best results of the other models.

**Table 1:** Performance of different models using data from 4 provinces.

Province and forecast period		Model						
		Historical average	AR^a^	ARIMA^b^	LSTM^c^	ST-GCN^d^	Ours (single graph)	Ours (multigraph)
		F1 score	F1 score	F1 score	F1 score, mean (SD)	F1 score, mean (SD)	F1 score, mean (SD)	F1 score, mean (SD)
**Beijing**								
	1-month prediction	0.679	0.742	0.734	0.750 (0.007)	0.777 (0.034)	0.811 (0.014)	0.822 (0.011)
	2-month prediction	0.675	0.741	0.720	0.744 (0.012)	0.737 (0.023)	0.785 (0.007)	0.812 (0.017)
	3-month prediction	0.674	0.733	0.664	0.743 (0.019)	0.724 (0.041)	0.768 (0.011)	0.805 (0.021)
**Zhejiang**								
	1-month prediction	0.484	0.597	0.558	0.551 (0.021)	0.651 (0.026)	0.648 (0.021)	0.679 (0.009)
	2-month prediction	0.471	0.562	0.474	0.501 (0.017)	0.604 (0.031)	0.630 (0.019)	0.660 (0.012)
	3-month prediction	0.457	0.531	0.404	0.441 (0.015)	0.544 (0.029)	0.603 (0.020)	0.645 (0.008)
**Shanxi**								
	1-month prediction	0.373	0.559	0.390	0.550 (0.022)	0.582 (0.045)	0.677 (0.011)	0.709 (0.013)
	2-month prediction	0.369	0.548	0.314	0.549 (0.027)	0.583 (0.039)	0.684 (0.015)	0.699 (0.019)
	3-month prediction	0.366	0.541	0.246	0.542 (0.017)	0.585 (0.043)	0.683 (0.012)	0.695 (0.017)
**Hebei**								
	1-month prediction	0.682	0.632	0.531	0.553 (0.018)	0.449 (0.027)	0.692 (0.005)	0.720 (0.006)
	2-month prediction	0.675	0.616	0.494	0.532 (0.016)	0.445 (0.048)	0.683 (0.012)	0.703 (0.010)
	3-month prediction	0.666	0.593	0.452	0.513 (0.020)	0.392 (0.033)	0.668 (0.007)	0.698 (0.012)

^a^AR: autoregressive.

^b^ARIMA: autoregressive integrated moving average.

^c^LSTM: long short-term memory.

^d^ST-GCN: spatial–temporal graph convolutional network.

#### Effect of Spatial Dependence

The results of the Beijing data set, using 4 different spatial graphs to represent spatial dependence between regions and multiple spatial graph fusion (Table 2), demonstrate that different spatial dependence affects prediction: single spatial dependence is not as effective as the fusion of multiple dependencies.

**Table 2:** Performance of models with different spatial dependencies.

Model type		F1 score		
		1-month prediction	2-month prediction	3-month prediction
**Single-graph**				
	Proximity	0.813	0.785	0.768
	Time series similarity	0.800	0.776	0.732
	POI similarity	0.797	0.705	0.741
	Exposure food similarity	0.813	0.756	0.743
Multigraph		0.822	0.812	0.805

#### Effect of External Features

Using the Beijing data set, the performance of models with external features is slightly better than those of models without external features for 1-, 2-, and 3-month predictions (Table 3), which demonstrates that the external features affect the trend of foodborne disease to some extent.

**Table 3:** Performance of models with or without external features.

Model type	F1 score		
	1-month prediction	2-month prediction	3-month prediction
External features	0.818	0.810	0.803
No external features	0.822	0.812	0.805

#### Effect of Attention Mechanism

For the Beijing data set, the removal of the attention mechanism in the spatial dimension or in the temporal dimension reduced the effectiveness of the model (Table 4). With the removal of the attention mechanism in the temporal dimension, as the prediction range increased, model performance decreased. This also confirms that, in the multistep prediction, the use of an attention mechanism can solve the distance problem in sequence dependence.

**Table 4:** Performance of models with or without an attention mechanism.

Model type	F1 score		
	1-month prediction	2-month prediction	3-month prediction
Spatial attention only	0.815	0.801	0.788
Temporal attention only	0.807	0.805	0.798
With attention mechanism	0.822	0.812	0.805

### Mapped Results

**Figure 5:**
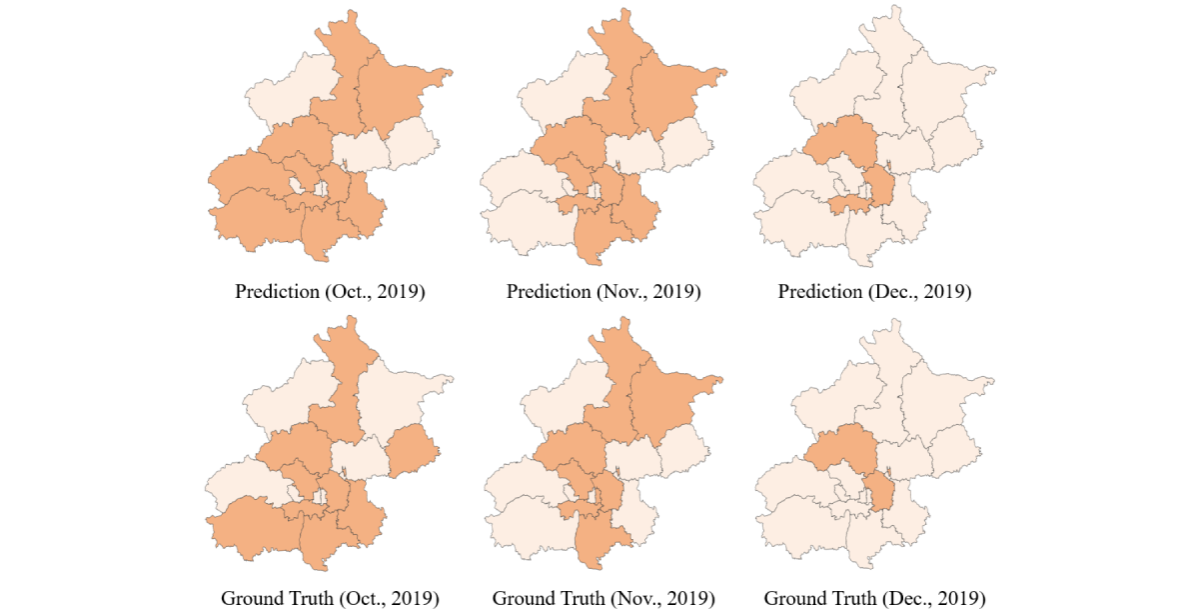
Case study 1: The first row displays the predictions and the second row displays ground truths for Beijing in October, November, and December in 2019.

We selected 3 consecutive months in the Beijing data set (October, November, and December 2019), for which we mapped the predicted values and the ground truths (Figure 5). Disease risks in most regions were correctly predicted, and only 1 or 2 regions had incorrect predictions for each prediction range. Incorrect predictions were often affected by the value of the surrounding region, which is also consistent with clustered outbreak characteristics of foodborne diseases. To a certain extent, this case suggests that our model is able to capture the spatial–temporal correlations between data and can provide accurate multistep prediction.

**Figure 6:**
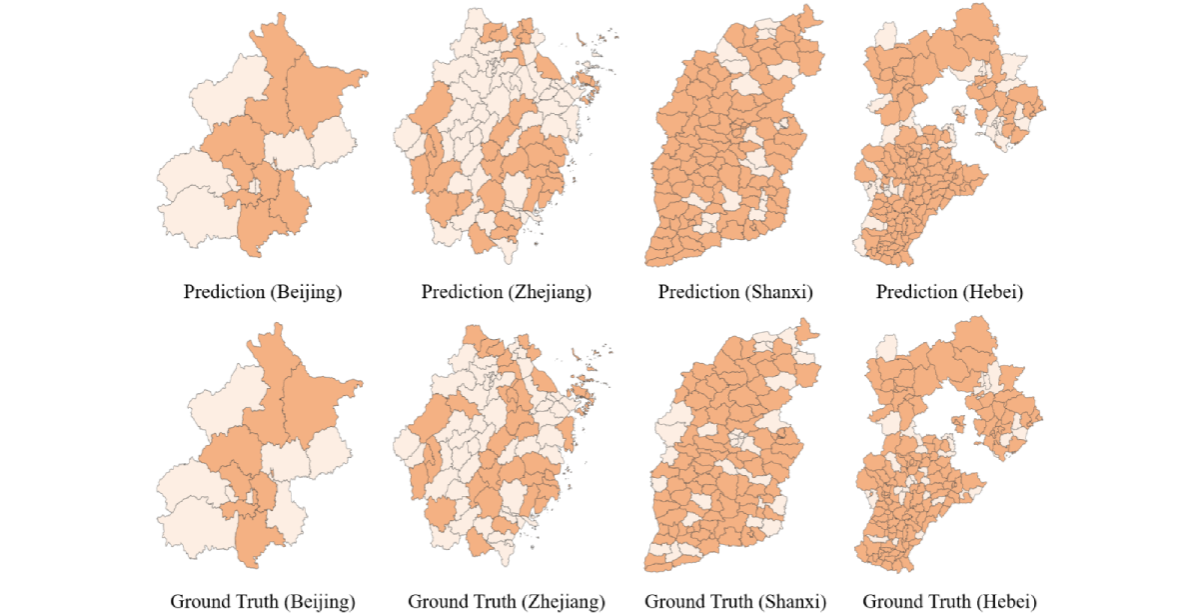
Case study 2: The first row displays predictions for Beijing, Zhejiang, Shanxi, and Hebei in November 2019, and the second row displays the ground truths for Beijing, Zhejiang, Shanxi, and Hebei in November 2019.

We use the same method to display the results of each province in November 2019 (Figure 6), demonstrating that our model can correctly predict disease risk in these 4 provinces to a large extent. Due to the difference in the number of counties and cities in each province, model prediction accuracies differed. Provinces with more subregions had more incorrect predictions. As in the previous case, most regions with incorrect predictions were the values of surrounding regions. In general, our model can achieve good results in predicting spatial–temporal foodborne disease risk and has a certain degree of robustness. It can achieve multistep disease risk prediction, which can provide more information for the prevention and control of foodborne disease.

## Discussion

### Principal Results

Our proposed model utilizes structural LSTM to model spatial dependence and temporal dependence in data and takes into account multiple spatial correlations rather than the single spatial proximity. We also incorporated external features and spatial–temporal attention mechanisms to refine the model. The model was validated using the real-world foodborne disease data sets.

The results demonstrate that our model performs better than other models, for the 4 provinces that we selected, in determining future foodborne disease risk. Our model with multiple spatial graphs achieved the best prediction results for all provinces and prediction ranges, and our model with a single graph achieved the second-best prediction results in most cases, which shows that compared to other prediction models, including statistical models and deep learning models, our method can model temporal and spatial dependence better.

We have a better understanding of the influence of each module of the model on prediction from experiments with spatial dependence, including external features, and including an attention mechanism. Each spatial dependence has a different effect on model prediction, and models that only use a single spatial dependence are not as effective as models that use multiple spatial dependencies. Models with external features will have more accurate risk prediction results; we also use the same method to conduct experiments to verify the influence of spatial–temporal attention on the model, and the spatial–temporal attention mechanism had a positive effect on the model. Mapped results demonstrate that our model is accurate, with long-term prediction advantages, and that our model is robust, meaning that it can be used for nationwide foodborne disease risk prediction. We found that most incorrect predictions are clustered (and predicted to be the value of a nearby area).

### Limitations

This study has certain limitations. First, due to the difficulty in obtaining multisource data and because model training takes a long time, we only selected 4 provinces (those with the best-quality data) to conduct experiments. Therefore, the experimental results may not be representative of all provinces in the country. In the future, we will conduct more experiments in more provinces to validate the model. Second, our model takes 4 spatial correlations into account, but real spatial correlations may be more complicated. Therefore, in the future, we will further analyze foodborne disease data and correlations with other data, to refine our model. Third, our model uses month as the temporal unit. Month-based risk prediction can better estimate long-term disease risk; however, the use of finer time-granularity disease risk prediction can provide more precise guidance for disease risk prevention and control disease risk prediction that uses smaller units can provide more comprehensive support for the prevention of foodborne diseases.

### Conclusions

We focused on foodborne disease risk prediction and proposed a multigraph structural LSTM spatial–temporal prediction model based on an encoder–decoder structure. Disease risk in each region in the future was considered to be influenced by the historical disease records as well as by disease risk in surrounding areas. Moreover, in addition to proximity in space, other spatial correlations that affect disease risk prediction were taken into account by using an adaptive multigraph fusion method to adjust the effect of spatial dependencies in different circumstances. We also added a spatial–temporal attention mechanism and external features to refine the model.

Applied to a real-world foodborne disease data set from Beijing, Zhejiang, Shanxi, and Hebei, the model’s performance was better than those of other models, and highest F1 score was 20% higher than the best results of the other models. Our model can better predict the risk of foodborne diseases in the future and can provide supporting data for risk assessment, prevention, and control of foodborne diseases.

In the future, we will evaluate our model in more provinces, consider more spatial correlations, with finer time granularity, and construct an interactive foodborne disease risk prediction system that can provide more intuitive and convenient supporting data for the prevention of foodborne diseases.

## Abbreviations

COVID-19: coronavirus disease 2019
LSTM: long short-term memory
POI: point of interest

## References

[1] Cliver D, Riemann H (2002). Foodborne Diseases.

[2] Oliver SP (2019). Foodborne pathogens and disease special issue on the national and international pulsenet network. Foodborne Pathog Dis.

[3] Swaminathan B, Barrett TJ, Hunter SB, Tauxe RV (2001). PulseNet: the molecular subtyping network for foodborne bacterial disease surveillance, United States. Emerg Infect Dis.

[4] McCabe-Sellers BJ, Beattie SE (2004). Food safety: emerging trends in foodborne illness surveillance and prevention. J Am Diet Assoc.

[5] Li W, Pires SM, Liu Z, Ma X, Liang J, Jiang Y, Chen J, Liang J, Wang S, Wang L, Wang Y, Meng C, Huo X, Lan Z, Lai S, Liu C, Han H, Liu J, Fu P, Guo Y (2020). Surveillance of foodborne disease outbreaks in China, 2003–2017. Food Control.

[6] Gallo M, Ferrara L, Calogero A, Montesano D, Naviglio D (2020). Relationships between food and diseases: what to know to ensure food safety. Food Res Int.

[7] Thakur M, Olafsson S, Lee J, Hurburgh CR (2010). Data mining for recognizing patterns in foodborne disease outbreaks. J Food Eng.

[8] Sadilek A, Kautz H, Silenzio V (2012). Predicting disease transmission from geo-tagged micro-blog data.

[9] Sadilek A, Kautz H, DiPrete L, Labus B, Portman E, Teitel J, Silenzio V (2017). Deploying Nemesis: preventing foodborne illness by data mining social media. AIMag.

[10] Effland T, Lawson A, Balter S, Devinney Katelynn, Reddy Vasudha, Waechter HaeNa, Gravano Luis, Hsu Daniel (2018). Discovering foodborne illness in online restaurant reviews. J Am Med Inform Assoc.

[11] Vilne B, Meistere I, Grantiņa-Ieviņa L, Ķibilds J (2019). Machine learning approaches for epidemiological investigations of food-borne disease outbreaks. Front Microbiol.

[12] Pan W, Zhao J, Chen Q (2015). Classification of foodborne pathogens using near infrared (NIR) laser scatter imaging system with multivariate calibration. Sci Rep.

[13] Xiao X, Ge Y, Guo Y (2015). Automated detection for probable homologous foodborne disease outbreaks.

[14] Neill D, Moore A (2004). Rapid detection of significant spatial clusters.

[15] Nogueira M, Greis N, Bassiliades N, Governatori G, Paschke A (2011). Rule-based complex event processing for food safety and public health. Rule-Based Reasoning, Programming, and Applications.

[16] Wu Y, Yang Y, Nishiura H (2018). Deep learning for epidemiological predictions.

[17] Wang L, Chen J, Marathe M (2019). DEFSI: deep learning based epidemic forecasting with synthetic information.

[18] Aramaki E, Maskawa S, Morita M (2011). Twitter catches the flu: detecting influenza epidemics using twitter.

[19] Akaike H (1969). Fitting autoregressive models for prediction. Ann Inst Stat Math.

[20] Box GEP, Pierce DA (1970). Distribution of residual autocorrelations in autoregressive-integrated moving average time series models. J Am Stat Assoc.

[21] Rumelhart DE, Hinton GE, Williams RJ (1986). Learning representations by back-propagating errors. Nature.

[22] Hochreiter S, Schmidhuber J (1997). Long short-term memory. Neural Comput.

[23] LeCun Y, Boser B, Denker JS, Henderson D, Howard RE, Hubbard W, Jackel LD (1989). Backpropagation applied to handwritten zip code recognition. Neural Comput.

[24] Defferrard M, Bresson X, Vandergheynst P (2016). Convolutional neural networks on graphs with fast localized spectral filtering. Proceedings of the 30th Annual Advances in Neural Information Processing Systems.

[25] Zhao L, Song YJ, Zhang C, Liu Y, Wang P, Lin T, Deng M, Li H (2020). T-GCN: A Temporal Graph Convolutional Network for Traffic Prediction. IEEE Trans Intell Transport Syst.

[26] Jain A, Zamir R, Savarese S (2016). Structural-RNN: deep learning on spatio-temporal graphs. Proceedings of the IEEE Conference on Computer Vision and Pattern Recognition.

[27] Chen D, Yang Y, Zhang Y, Yu W (2020). Prediction of COVID-19 spread by sliding mSEIR observer. Sci China Inf Sci.

[28] Li Y, Zou X (2016). Identifying disease modules and components of viral infections based on multi-layer networks. Sci China Inf Sci.

[29] Zhang J, Zheng Y, Qi D, Li R, Yi X, Li T (2017). Predicting citywide crowd flows using deep spatio-temporal residual networks. Proceedings of the AAAI Conference on Artificial Intelligence.

[30] Guo S, Lin Y, Feng N, Song C, Wan H (2019). Attention based spatial-temporal graph convolutional networks for traffic flow forecasting. AAAI Conference on Artificial Intelligence.

[31] Yu B, Yin H, Zhu Z (2018). Spatio-temporal graph convolutional networks: a deep learning framework for traffic forecasting. Proceedings of the 27th International Joint Conference on Artificial Intelligence.

[32] Wang B, Luo X, Zhang F (2018). Graph-based deep modeling and real time forecasting of sparse spatio-temporal data.

[33] Kim Y, Wang P, Mihaylova L (2019). Structural recurrent neural network for traffic speed prediction.

[34] Ramos J (2003). Using TF-IDF to determine word relevance in document queries. Semantic Scholar.

[35] Foodborne disease surveillance and reporting system. China National Center for Food Safety Risk Assessment.

